# Multi-peril pathogen risks to global wheat production: A probabilistic loss and investment assessment

**DOI:** 10.3389/fpls.2022.1034600

**Published:** 2022-10-31

**Authors:** Yuan Chai, Senait Senay, Diana Horvath, Philip Pardey

**Affiliations:** ^1^ Department of Applied Economics, University of Minnesota, St. Paul, MN, United States; ^2^ GEMS (Genetics x Environment x Management x Socio-economics) Informatics Center, University of Minnesota, St. Paul, MN, United States; ^3^ Department of Plant Pathology, University of Minnesota, St. Paul, MN, United States; ^4^ 2Blades, Evanston, IL, United States

**Keywords:** wheat, fungal diseases, biotic risks, disease losses, R&D investment

## Abstract

Crop diseases cause significant food and economic losses. We examined the joint, probabilistic, long-term, bio-economic impact of five major fungal pathogens of wheat on global wheat production by combining spatialized estimates of their climate suitability with global wheat production and modeled distributions of potential crop losses. We determined that almost 90% of the global wheat area is at risk from at least one of these fungal diseases, and that the recurring losses attributable to this set of fungal diseases are upwards of 62 million tons of wheat production per year. Our high-loss regime translates to around 8.5% of the world’s wheat production on average—representing calories sufficient to feed up to 173 million people each year. We estimate that a worldwide research expenditure of $350-$974 million (2018 prices) annually on these five fungal diseases of wheat, let alone other pathogens, can be economically justified, equivalent to 2 to 5 times more than the amount we estimate is currently spent on *all* wheat disease-related public R&D.

## Introduction

Crops are food not just for people and animals, but also for numerous microbial pathogens and insect pests. Often the impacts of a specific pathogen on a particular plant host are examined individually, but the reality is that agro-ecological regions more typically have conditions favorable for multiple pathogens. For many human diseases such as the most recent SARS-CoV2 outbreaks, access to real-time data on the movement and variation of pathogen strains has helped prioritize responses to the pandemic. However, the data on most plant diseases are fragmented and often out of date, making it difficult to accurately assess the impacts of crop diseases and appropriately allocate resources for mitigation efforts. In this study, we use a probabilistic, multi-peril approach to examine the worldwide risks and impacts of the most significant fungal pathogens affecting wheat, and explore their policy implications in terms of the economically justifiable research and development investments globally to mitigate the wheat losses attributable to these pathogens.

Wheat is an especially critical component of global food supplies. This single crop accounts for about one-fifth of the total calories and total protein consumed by the planet’s 7.9 billion people each and every year (FAO, 2020). United Nations (2022) projections have global population approaching 10 billion people by 2050. This inexorably growing population, coupled with increasing per capita incomes, will continue to push the global demand for food ever higher (Pardey et al., 2014). Against these stark food consumption futures is an equally stark agricultural production reality. Crop yields are intrinsically variable due to fluctuating and sometimes extreme weather, and doubly so given the losses from a range of biological threats, i.e., diseases, insects, animals, and weeds—collectively “pests,”—that can compromise yields and reduce quality, taste, nutrition, and food safety (see, e.g., Reynolds et al., 2007).

The pests that impact wheat crops are representative of the problem, which results in significant, albeit temporally and spatially variable, damage—ostensibly accounting for 10-50% (on average) of wheat crop losses worldwide (e.g., Oerke, 2006; Savary et al., 2019). In the U.S., $209 million was paid out to wheat farmers on insurance claims for crop losses attributable to insects, diseases, and weeds between 2010-2020 (USDA-RMA, 2021, authors’ calculation), even *after* farmers had taken preventive steps by applying herbicides to 71% of their wheat acreage, fungicides to 30%, and insecticides to 7% of the cropped area (in 2017) (USDA-NASS, 2018, authors’ calculations). For smallholder farmers in developing countries, crop pest losses are also likely large, especially given the limited uptake of modern seed varieties and the even more limited use of agricultural chemicals that can mitigate these losses (Sheahan and Barrett, 2017; Pardey et al., 2022). Furthermore, climate change is amplifying these issues by increasing plant stress and expanding the natural ranges of pathogens, as well as the geographical risk exposure to the consequences of crop pests (Bebber et al., 2013). Chaloner et al. (2021) projected that infection risks from 80 plant pathogens are likely to increase at higher latitudes in the future, exerting even greater burden for securing global crop production under climate change challenges.

The substantial and continuing economic loss and damage to livelihoods from crop pests present a *prima facie* case for investing in innovations that address these pervasive and perennial crop-pest problems. Determining how much to invest and how to prioritize investments among the many pests that affect wheat (and other crops) is difficult. Getting a sense of the magnitude of the losses is key to estimating the amount of investment that a hard-nosed economic assessment would support to avoid these losses. However, the prior bio-economic evidence is patchy—particularly at geographical scale (such as a country, and especially worldwide)—and is less useful for making strategic innovation investment choices. Losses attributable to pests vary seasonally and geographically. Where credible long-run loss data exist—e.g., Pardey et al. (2013) in the case of wheat stem rust losses affecting U.S. farmers for over a century—they reveal that even for this problematic pest, in numerous years the losses are negligible. Thus, it is an overstatement to consider losses for a particular pest in a given year and locale (especially a localized extreme loss) as being representative of the longer-run average annual losses at scale (say for a country or the world). Furthermore, farmers are subject to the yield-reducing effects of multiple pests, and so estimates of the combined losses arising from these multiple threats is required to properly calibrate the overall magnitude of the investments justified to deal with these multi-peril pest problems.

In this study we examined the economic impacts of five fungal pathogen threats to wheat by taking into consideration the complex interactions among environment-pathogen-host in disease-related crop losses. We focused on estimating potential losses caused by five fungal diseases, namely stem rust (*Puccinia graminis*), stripe rust (*Puccinia striiformis*), leaf rust (*Puccinia triticina*), fusarium head blight (FHB) (*Fusarium graminearum*), and septoria tritici blotch (STB) (*Zymoseptoria tritici*), that afflict wheat producers in rich and poor countries alike (see, e.g., Figueroa et al., 2018). We combine spatially-explicit estimates of each pathogen’s climate suitability with global wheat production and modeled potential loss distributions to jointly assess the long-term impact of this crop disease complex and their policy implications. Our novel, multi-peril pest approach accounts for the location-to-location and season-to-season variation in pest-related damage to crops. We consider the risk profile faced by farmers worldwide from this portfolio of pests and extend the probabilistic loss methodology—hitherto used on a pest-by-pest basis (e.g., Pardey et al. (2013) for stem rust, Beddow et al. (2015) for stripe rust, and Chai et al. (2020) for leaf rust)—to assess the overall losses jointly attributable to the five fungal diseases. We then used the losses to estimate the implied research and development (R&D) investments worldwide that are economically justified to mitigate these losses under both high- and low-loss regimes.

## Results

### Global wheat vulnerability to fungal diseases

Wheat was planted on 219 million acres globally in 2020, equal to about one-third of the world’s total area for cereal agriculture. Strikingly, we find that 80% or more of that global wheat area is at risk from four fungi: FHB, and leaf, stripe, and stem rust infection (**Table 1**). In addition to these four pathogens, around half the wheat area is climate-suitable for STB.

**Table 1:** Share of regional wheat growing areas at risk from fungal disease.

	Harvested Wheat area		Wheat fungal disease suitable area share				
Region	Amount	Global share	FHB	Leaf Rust	Stripe Rust	Stem Rust	STB
	*(million hectares)*	*(percent)*	*(percent)*				
**Developed World**	**138.0**	**57.6**	**80.3**	**80.6**	**81.5**	**73.2**	**56.9**
Former Soviet Union	51.9	21.7	54.4	54.7	56.6	44.3	22.3
Western Europe	50.4	21.0	97.0	97.2	98.6	96.8	79.6
North America	25.3	10.5	95.3	95.4	92.0	85.4	83.5
Australasia	10.4	4.4	82.2	83.2	87.3	66.8	43.8
**Developing World**	**101.6**	**42.4**	**98.9**	**94.8**	**80.0**	**92.5**	**50.7**
Asia	89.4	37.3	99.0	94.6	78.0	92.2	46.1
Latin America	9.4	3.9	98.6	97.1	92.7	94.7	80.5
sub-Saharan Africa	2.8	1.2	97.1	94.1	90.6	93.7	74.5
**World**	**239.6**	**100.0**	**86.8**	**85.5**	**80.9**	**79.9**	**54.7**

Source: Developed by authors.

For ease of exposition, we aggregated some of the Beddow et al. (2013) zones when reporting the results in this table. Specifically, the Latin America and the Caribbean (LAC) figures represent an aggregation of the estimates formed for the Central America & Caribbean, Andean and Eastern South America zones. Sub-Saharan Africa (SSA) is an aggregation of the East Central Africa, West Central Africa and Southern Africa zones. Asia groups together the Southeast Asia, Southwest Asia and Northeast Asia zone estimates. Western Europe includes all countries on the European Continent from the North Africa & West Europe and Eurasian zones. FSU is the Former Soviet Union countries.

Rows with values in bold represent the aggregated values for countries in "Developed World", "Developing World" and all "World", respectively.

Rows with values in bold represent the aggregated values for countries in “Developed World”, “Developing World” and all “World”, respectivelyThese pathogens co-evolved over centuries in tandem with wheat, and thus it is of little surprise that the same climate also sustains these fungi (Thompson and Burdon, 1992). However, wheat has moved well beyond its ancestral center of origin in the Fertile Crescent that spans modern countries of Israel, Jordan, Syria, Lebanon, eastern Turkey, western Iran, and northern Iraq (see, e.g., Khoury et al., 2016). It now grows in latitudes stretching from 67° north in Norway, Finland, and Russia to 45° south in Argentina (Joglekar et al., 2016; Yu et al., 2020). Different varieties of wheat are more or less suitable for different climatic conditions, and similarly fungal diseases vary by climate and locale. These spatial sensitivities are reflected in the regional data summarized in **Table 1**. Our analysis shows that even in more recently farmed or minor wheat growing areas, all five diseases pose a significant threat, with many capable of occurring on more than 90% of the wheat areas in sub-Saharan Africa and Latin America. The geographic extent of wheat areas deemed climate-suitable for fungal infections is generally of comparable magnitude in North America and Western Europe. The Former Soviet Union countries collectively account for about one-fifth of the world’s wheat area, much of it in more northerly latitudes, however in this region the climate suitability for all five fungal diseases is more restricted. Our results suggest that the most significant risk to the world supply of wheat comes from production that occurs in Asia. This region accounted for 30.9% of the world’s wheat harvested area in 2020, and wheat there is vulnerable to infection from several fungi, with climate-suitable area shares ranging from 92.2%-99.0% for stem rust, leaf rust and FHB diseases.

**Figure 1:**
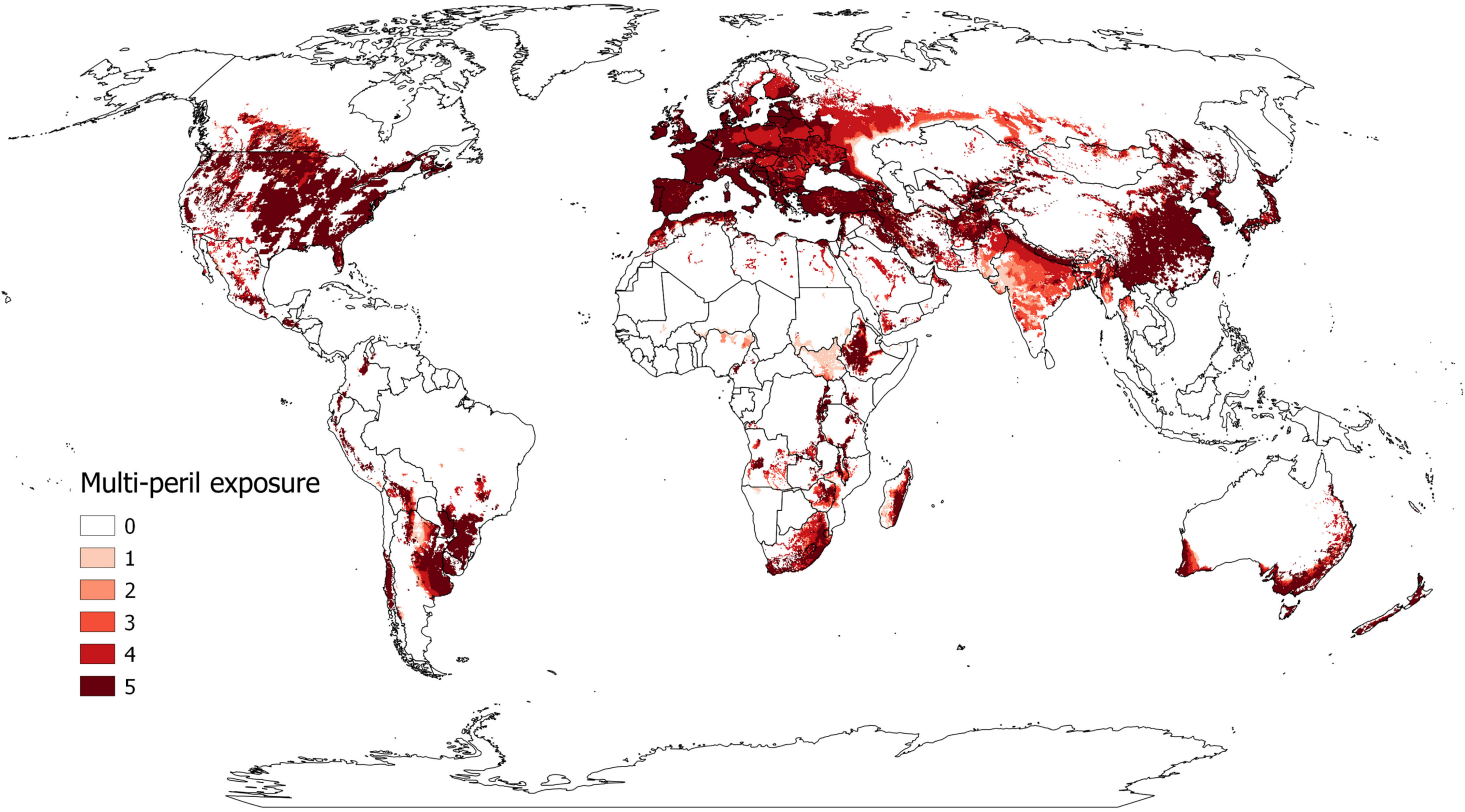
Modeled global climate-based multi-peril exposure for selected wheat fungal diseases. This map reports the climate-based multi-peril exposure (pest suitability count) of modeled CLIMEX annual growth indexes (GI) for the following wheat fungal diseases: stem rust, stripe rust, leaf rust, FHB, and septoria tritici blotch. The map shows the count of disease suitability (i.e., GI > 5) within the global wheat production areas reported by the Spatial Allocation Model (SPAM) (Yu et al., 2020).

Within the spatial extent of global wheat production, **Figure 1** maps 10 arc minute pixelated spatial grids with climates that can sustain various fungi for at least one life cycle of the disease, differentiating between those pixels that are climate suitable for none, one, and so on up to five of the pests. The shading in **Figure 1** thus represents the multi-peril risk exposure for the five wheat fungal diseases at each location. The darker the shading, the more pests that can be sustained at a given pixel. Most of the map tends toward the darker end of the spectrum, consistent with the summary of this co-suitability phenomenon in **Supplementary Table S1**. Starkly, only 11.4% of the world’s wheat area occurs in locales that none of the five pests find climate suitable. More than 75% of the world’s wheat will sustain at least four of the pests, and more than half (54.0%) of the area is susceptible to all five diseases.

Notably, the more-developed countries grow their wheat in locations that tend to have less multi-peril risk exposure than countries throughout the developing world. Nonetheless, developed countries are by no means free of these risks. We estimate that well over half (56.4%) of the wheat area in developed countries is threatened by all five fungi. In the developing world, wheat crops in sub-Saharan Africa and Latin America are especially vulnerable to infestations from multiple fungi; more than 90% of the wheat area in both regions are at risk from at least four fungi, while in Asia 92% of the area is vulnerable to at least three pests. Moreover, many of the poorer wheat producers in these areas have less access to resistant varieties, fungicides, and information about best management practices.

### Global wheat loss estimates

**Figure 2:**
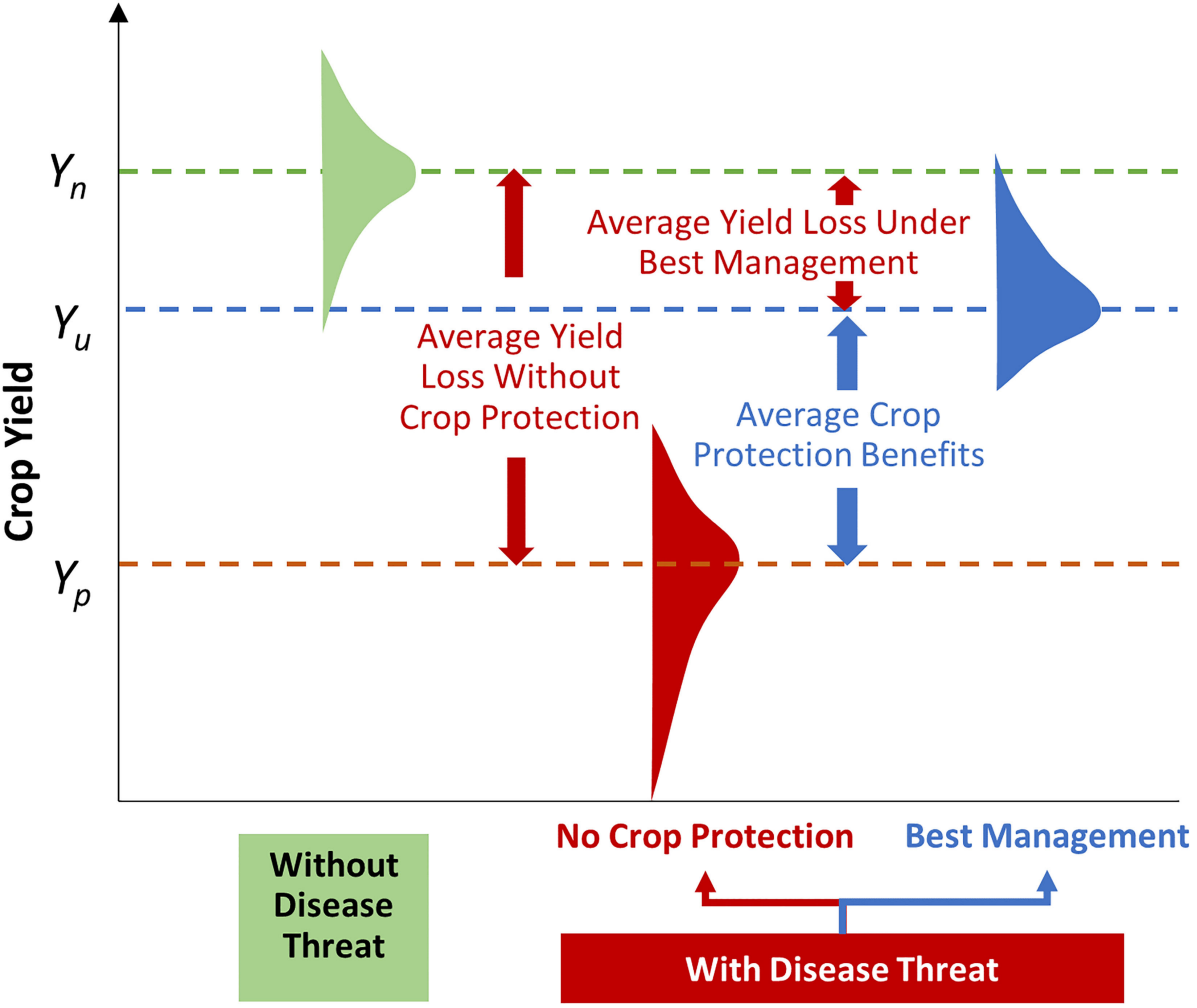
Raising crop productivity and derisking farmers’ yields. The green distribution reflects variation in (field-scale) crop yields around an average yield, *Y_n_*, that is free of any pest threats. Here the distribution in yields represents the effects of season-to-season variation in local weather. *Y_p_* represents the average yield for the same crop in the same agroecology, but now yields vary in response to both local weather and pests. This red-shaded distribution has a long downside tail stretching to zero to indicate low probability but nonetheless severe (and in rare cases, total) loss events arising from a pest infestation. The difference in yields (*Y_p_ – Y_n_*) represents the losses, *on average*, attributable to pest threats. Through deployment of best pest management and mitigation practices, the yield losses due to crop pests can be restored, and if *fully effective*, would raise yields (or conversely reduce unit costs of production), on average, from *Y_p_* to *Y_n_*. A *cost-effective* approach to dealing with crop pests is unlikely to fully restore yields (and thus household food and nutrition access, and perhaps farm incomes) *on average*, or eliminate yield risk entirely. Rather it would involve protecting yields and reducing risks up to the point that the marginal (or incremental) benefits equals the marginal costs of ameliorating the crop damage consequences of pests. This is indicated by the shift from the red to blue yield distribution profiles, where average yields are increased from *Y_p_* to *Y_u_* (the average yield under the no-disease-threat regime), that is largely, but not fully, restored and the dispersion in yields around the average is reduced. This reduction in risk is visually represented in terms of the more peaked yield distribution (blue) when farmers use pest-reducing innovations versus when they do not (red), such that reducing the variability in yields de-risks cropping for farmers and others in the supply chain.

The occurrence and magnitude of crop losses due to disease depends on several factors. Locations that are climate-suitable for a particular pest certainly put farmers at risk from that pest, but it does not necessarily mean in any particular cropping season they incur crop losses from it. The odds of a crop being infected, the severity and timing of an infection, and the dispersal mechanisms that can spread the disease well beyond its initial site of infection are part of the complex epidemiological processes that affect the geographic extent and magnitude of pest-induced crop losses. **Figure 2** provides a conceptual (and empirically tractable) visualization of the effects of pests on crop yields that is especially useful for impact assessment purposes. The with- and without-disease threat construct in this framing provides the basis for the counterfactual underpinnings of our loss assessment approach, which in principle can be applied at any spatial scale (be it an experimental plot, a farm field, a country, etc.). In addition, the loss likelihood functions we estimated based on reported historical observations (see Methods and **Supplementary Figure S1**, and visualized as the respective green, red and blue yield distributions in **Figure 2**) capture the highly variable nature of the crop losses associated with a particular disease from year-to-year at any particular location. Differences in each disease’s epidemiological characteristics result in different modeled yield loss distributions. Climate has played a role in the historic year-to-year variability, just as it does in the location-to-location variability of disease-induced crop losses, thus, this analysis factors in impacts of climate variability. Facing uncertain climate change challenges, future impacts may be even more variable.

In our analysis, we use historical data to derive high- and low-loss regimes. We introduced two counterfactual regimes to help bound the range of likely global (or regional) losses, by applying these constructs on a pixelated basis at scale, across more- or less-advanced wheat farming practices throughout the world. The notes to **Figure 2** describe details of the (typically unobserved) “without” and (observed) “with” disease threat counterfactual construct. In farmers’ fields, all factors in the environment-pathogen-host disease triangle can affect the actual loss experienced by farmers. In constructing our high- and low-loss regimes, the goal is not to predict the loss that will occur in any particular season at a particular location. Rather, our assumption is that, within the wheat areas that are climatically suitable for these wheat fungal diseases, the *probabilistic loss distribution* of wheat fungal diseases derived from long-term historical data across the U.S. can serve as proxy loss scenarios for wheat growers elsewhere in the world.

Climate conditions, disease pressure, and wheat production practices vary greatly across locations. These result in spatially-variable wheat yields that are intrinsic to the spatially-explicit loss estimation procedure we describe below. To reflect variation in the use of genetics and fungicides and other factors that can influence the magnitude of the *percentage* losses (relative to spatially-variable yield *levels*) we use our yield-loss *distribution* evidence to construct both high- and low-loss regimes that plausibly bound our probabilistic loss estimates. Our high-loss regime represents a situation where disease pressures are high and/or farmers failed to implement effective disease management practices, while the low-loss regime represents a situation where disease pressures are low and/or well-managed in farmers’ fields. While our spatially-sensitive, probabilistic estimation methodology can be applied at different spatial scales (fields vs regions vs globally) spanning different time periods (within season or spanning multiple seasons, past or future), in this instance we use longer-term (historical) data to gain a bounded and strategic sense of the loss profiles at scale for each of the five fungal diseases separately and in aggregate to guide longer-term investment decisions concerning crop breeding or other methods to mitigate and manage these crop loss risks.

**Table 2** summarizes all this loss estimation complexity into three policy and investment related metrics—namely, the likely quantitative loss in global wheat production associated with each of the diseases one-by-one (columns 2-6), and the diseases as a multi-peril complex of diseases (column 1); the production losses expressed in U.S. dollar values (2018 prices); and the share of average annual wheat production likely lost to each of the diseases and the five diseases in total.

**Table 2:** Estimates of global annual losses in wheat production from fungal diseases.

	Total	FHB	STB	Stem Rust	LeafRust	StripeRust
	(1)	(2)	(3)	(4)	(5)	(6)
**Production loss (million metric tons)**						
***Low-loss regime***						
Mean	24.3	16.9	1.9	0.9	3.6	1.0
Range	(20.7-29.5)	(13.5-21.7)	(1.3-2.7)	(0.7-1.2)	(3.4-3.9)	(0.3-2.5)
***High-loss regime***						
Mean	62.0	28.5	10.6	9.9	8.9	4.1
Range	(56.4-70.0)	(23.4-35.9)	(9.7-11.7)	(8.7-11.6)	(8.2-9.8)	(3.5-4.9)
**Production loss (billion US$, 2018 prices)**						
***Low-loss regime***						
Mean	4.2	2.9	0.3	0.2	0.6	0.2
Range	(3.6-5.1)	(2.3-3.8)	(0.2-0.5)	(0.1-0.2)	(0.6-0.7)	(0.05-0.4)
***High-loss regime***						
Mean	10.8	4.9	1.8	1.7	1.5	0.7
Range	(9.8-12.1)	(4.1-6.2)	(1.7-2.0)	(1.5-2.0)	(1.4-1.7)	(0.6-0.8)
**Percentage of global average annual wheat production**						
***Low-loss regime***						
Mean	3.3	2.3	0.3	0.1	0.5	0.1
Range	(2.8-4.0)	(1.8-3.0)	(0.2-0.4)	(0.1-0.2)	(0.47-0.53)	(0.04-0.3)
***High-loss regime***						
Mean	8.5	3.9	1.4	1.4	1.2	0.6
Range	(7.7-8.5)	(3.2-4.9)	(1.3-1.6)	(1.2-1.6)	(1.1-1.3)	(0.5-0.7)

Source: Developed by authors.

Table entries represent estimates of average annual losses per year during the period 2020-2050.

Range (in brackets) represents the values between 5%-90% probability of loss.

We estimate that the *average annually recurring* loss of global wheat production from this complex of five fungal diseases lies in the range of 24.3 to 62.0 million metric tons, depending on the counterfactual loss regime. The intrinsic year-to-year variability in losses associated with crop pests and diseases is masked by the annual averages, but are revealed in the range entries. Thus, for example, while the high-loss regime projects average losses of 62.0 million metric tons per year, we estimate there is a 90% chance these global annual losses will exceed 56.4 million metric tons on average over the period 2020-2050, and a 5% chance the losses could be as high as 70.0 million metric tons per year. In dollar terms the overall losses are sizable, ranging from an annual average of $4.2 billion (2018 prices) worldwide in the low-loss scenario to $10.8 billion in the high-loss scenario. With estimated global wheat production projected to total $127.0 billion per year on average during 2020-2050, our estimates translate to annual losses that constitute between 3.3 and 8.5% of global wheat production.

**Figure 3:**
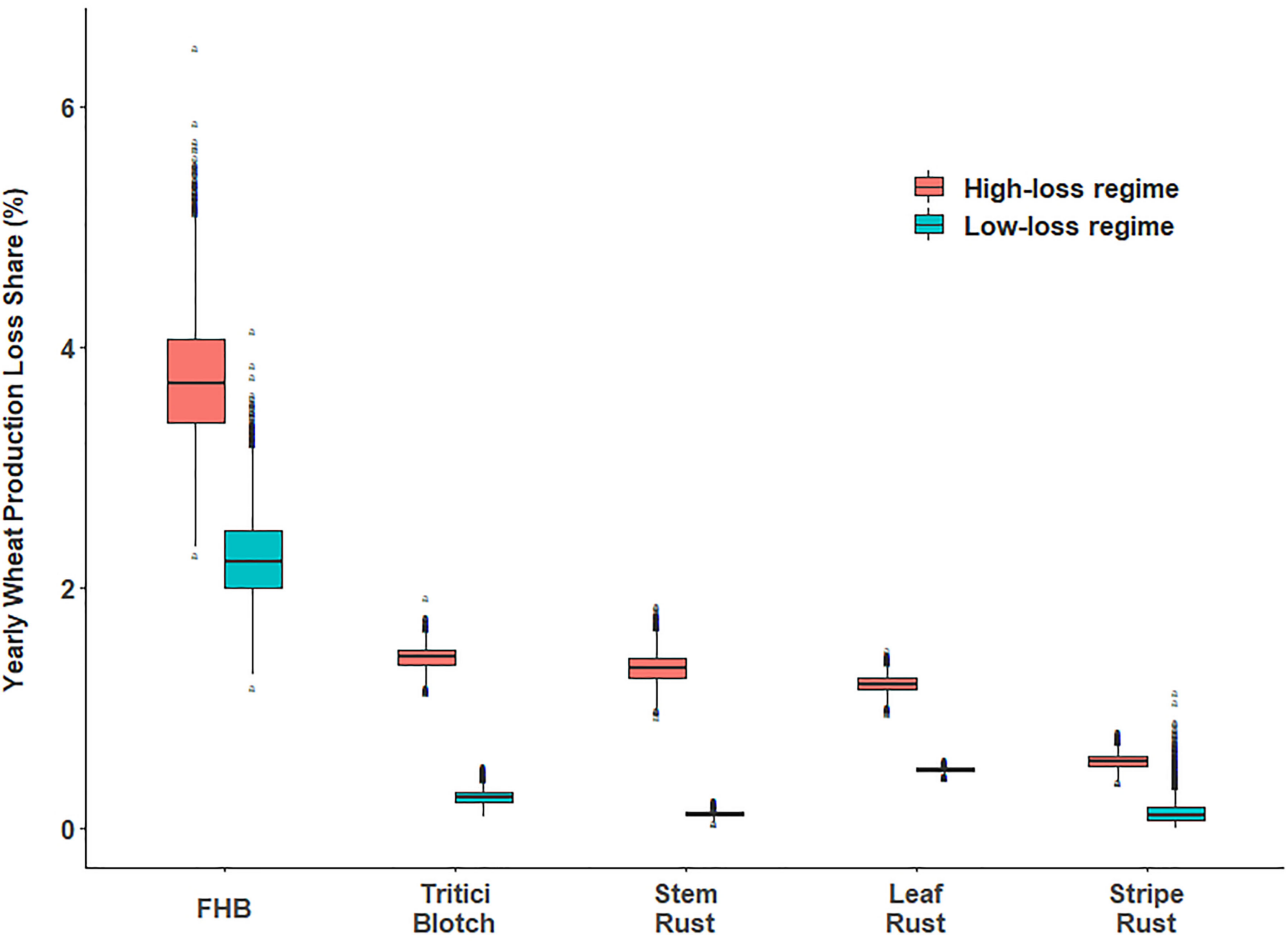
Distribution of simulated global yearly wheat production loss percentage attributable to selected wheat fungal diseases. Note: For the box plot, the lower and upper hinges correspond to the first and third quartiles (the 25th and 75th percentiles) with the middle line corresponds to the median from the distribution of yield losses. The upper (lower) whiskers extend from the upper (lower) hinge to the largest (smallest) value no further than 1.5 times of the inter-quartile range (distance between the first and third quartiles). Outliers are marked by black dots.

Complementing **Table 2**, **Figure 3** also provides information on the losses attributable to each of the fungal diseases. Complicating factors, the rank ordering of crop damages associated with each disease is sensitive to the counterfactual loss regime under consideration. For example, based on these estimates, FHB stands out as the dominant source of wheat losses in both the high- and low-loss regime. STB and stem rust are the next most damaging diseases. Under the high-loss regime, STB, stem and leaf rust have comparable loss profiles, whereas for the low-loss regime leaf rust slightly dominates the STB loss profile while stem rust losses are on par with those observed for stripe rust.

### The investment bottom line

Because R&D for innovation and the development of resilient crop varieties is an established and environmentally sound approach to avert crop losses from disease, the development of effective breeding solutions hinges on the question “*how much should be spent on innovative efforts to mitigate the crop losses attributable to fungal diseases of wheat*?” A defensible answer to this question requires recognizing the opportunity cost of these funds. That is, funds spent, say, on wheat fungal R&D cannot be spent on other types of R&D, or other means of ameliorating the crop losses due to fungal infections. Our investment estimation method explicitly factors in these opportunity costs by using the concept of the Modified Internal Rate of Return (MIRR) to calculate *the economically justifiable* R&D investments for the joint control of these five wheat fungal diseases.

**Figure 4:**
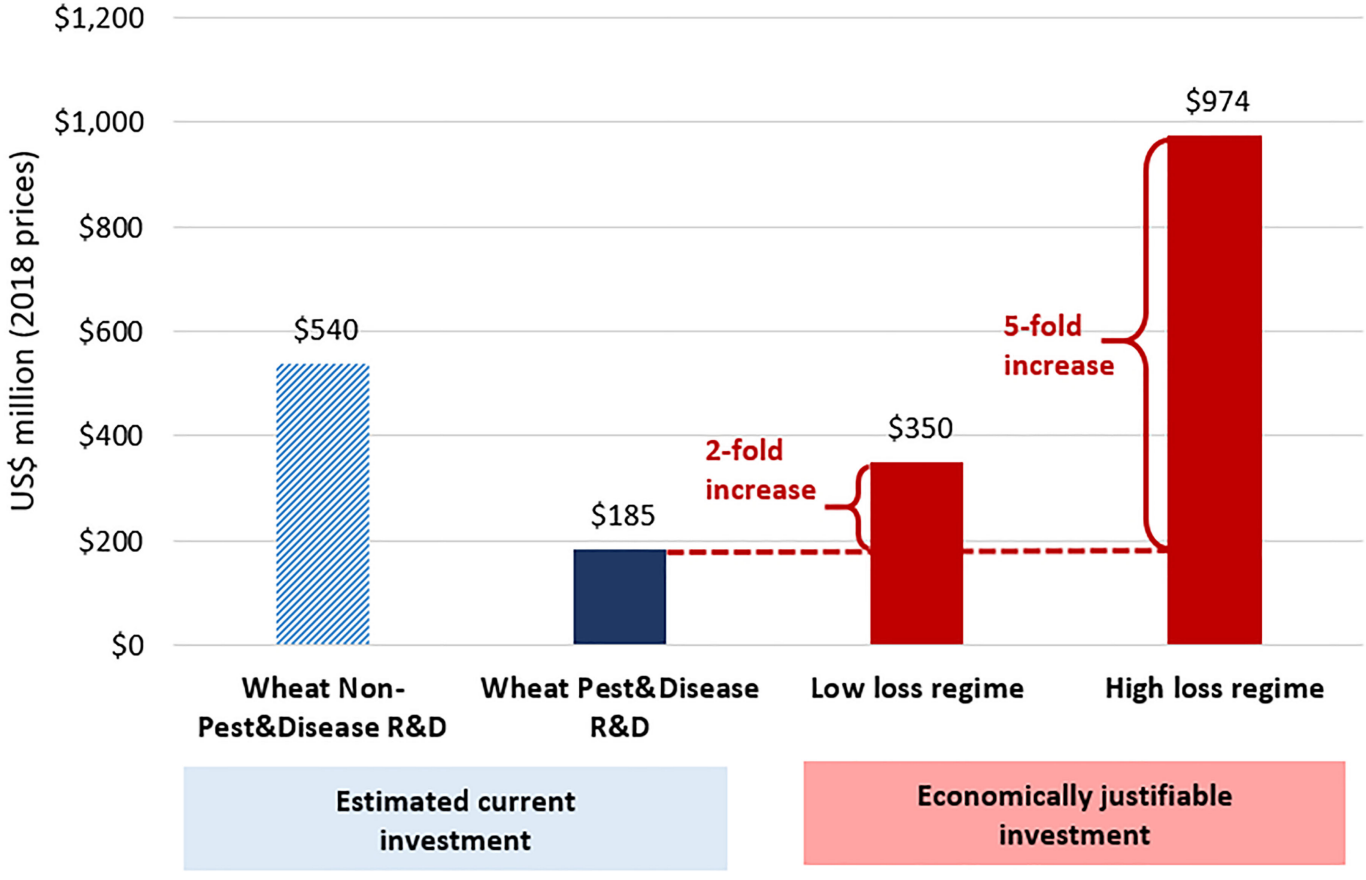
The Investment Bottom Line. All dollar estimates represent average annual values expressed in 2018 prices.

We estimate that to achieve a 10% per year return on investment (the economic benchmark for public agricultural R&D worldwide, according to findings by Rao et al., 2020) would require investing between $350 million (based on the low-loss regime) and $974 million (high-loss regime) on research for just these five fungal diseases of wheat, let alone the other biotic threats affecting wheat and other crops (**Figure 4**, right-hand column). For comparison, the two left-hand bars in **Figure 4** shows the estimated annual investment in public wheat research worldwide averaged over the period of 1980-2015 was $540 million per year for “non-pest & disease” R&D and $185 million per year for “pest & disease” related research, both in 2018 prices. Thus, our estimate implies that current spending on agricultural R&D for crop disease resistance is woefully inadequate and justifies a significant increase in funding on disease-related R&D for wheat by at least 2- to 5-fold.

## Discussion

Fungal diseases have substantial negative consequences for global wheat production. Large shares of the world’s wheat growing area are at risk from infection by each of the fungi, and the multi-peril risk is high, with around 75% of the area at risk of infection from at least four of the five fungal diseases in this study. The multi-peril risks are particularly high in Latin America and even more so in sub-Saharan Africa. Although sub-Saharan Africa produced only 1.1% of the world’s wheat crop in 2019, that year it accounted for 5.4% of worldwide wheat consumption. It is also where large numbers of the world’s poor and food insecure people now reside, and increasingly so in the decades ahead if we fail to promote the region’s economic growth in general, and agricultural growth in particular. Here we estimate losses of upwards of $10.8 billion (2018 prices) on average, year in and year out for just this one crop and the specific set of biological threats we evaluated. Preventing these losses could provide sufficient calories to feed an additional 173 million people every year (for the high-loss regime), assuming per person calorie consumption at the 2018 global average rate reported by the U.N.’s Food and Agricultural Organization (2001 and 2021). Fungal and other pathogens also cause significant losses in other major food crops, such as corn, rice, and potato (Ristaino et al., 2021).

There is a large amount of economic literature on the rates-of-return to agricultural R&D. Rao et al. (2020) reviewed 3,426 estimates taken from 492 different studies and reported an overall benefit-cost ratio of 10:1 on average (i.e., every dollar invested in agricultural R&D returned a stream of benefits with a present value of 10 dollars). A benefit-cost ratio significantly greater than 1:1 indicates that governments would have profited society by doing more agricultural R&D, compared with investment opportunities normally available to them. Alston et al. (2020, p. vi) argued that the totality of the considerable evidence on the size and nature of the payoffs, and the potential for future payoffs (see, e.g., Alston and Pardey, 2021) “…supports at least a doubling of the overall investment in agricultural R&D performed both in national and international agencies.” Our results support a 2- to 5-fold increase in (public) R&D to mitigate fungal wheat pathogens, which at face value also implies placing a greater emphasis on reducing the risks associated with crop pests within the expanded portfolio of wheat research.

While the private sector accounts for an increasing share of agri-food R&D in aggregate (Pardey et al., 2016), the private presence is comparatively small in many low- and middle-income countries. Moreover, wheat improving research worldwide still has a significant public presence. For example, data underlying Chai et al. (2022) study of all the commercially grown wheat varieties in the U.S over the past century reveals that a large share of these varieties were bred by public agencies, and plant varietal improvement in Canada is still a largely public affair (Gray et al., 2017). And while the private sector has made significant in-roads to wheat breeding in Europe and the U.K. (see, e.g., Galushko and Gray, 2014), in Australia the shift towards privately-led crop improvement still involves complex collaborative research, funding and even co-ownership arrangements with public entities (Alston and Gray, 2013). Nonetheless, much of the fundamental and longer-term R-gene discovery and more basic research related to wheat diseases is conducted by public non-profit agencies throughout the world.

Other important investment questions involve who should pay for this increase in R&D funding, and how. Alston and Pardey (1996 and 2021) review many of the economic issues involved in these types of decisions, but from a global perspective, the bottom line is that the entire world’s wheat crop is highly vulnerable to these fungal diseases, so all the world’s wheat producers and consumers share in the benefits from dealing with these diseases. Thus, irrespective of whether the research is performed by national or international agencies, the funding is generating wheat innovations that have global collective value. And, similar to the COVID crisis still afflicting human health worldwide, crop pests that undermine plant health are capable of travelling great distances through wind patterns and *via* international trade and travel. Thus, solving the problem for, say, wheat stem rust infestations that impact farmers in the U.S. or Africa, means that solutions can be deployed for farmers elsewhere in the world. From an economic perspective, the R&D funding should be directed to the innovative efforts that are likely to deliver effective fungal resistance technologies the fastest and cheapest. However, given proper attention to technology access and use details, particular governments or donor agencies can be assured that funding this type of disease resistance research, even if the research is carried out by institutes or individuals located elsewhere in the world, will bear positive returns to local producers and consumers given the worldwide nature of many disease problems: a classic case of doing well by doing good (Tribe, 1991).

Of course, for these innovations to find their way onto farmers’ fields requires much more than investing in the (pre-breeding) R&D required to produce durable disease resistance solutions. In stark contrast to COVID vaccine solutions that have worldwide applicability, genetic technologies that confer crop disease resistance must be bred into a myriad of locally adapted wheat varieties that are best suited to the diverse agroecological environments in which the crop is grown (see, e.g., Chai et al., 2022). Crucially, it is those myriad varieties, safeguarded with disease resistance, that must then find their way into the hands of the millions of farmers who grow the crop. Thus, complementary investments in rural infrastructure, epidemiological tracking of pest outbreaks, education, and timely and accurate information to farmers, input suppliers and crop breeders (who all play complementary roles in mitigating the impacts of reoccurring or newly emerging pest outbreaks) are crucial components of the innovation package required to achieve durable crop diseases resistance on the ground.

## Methods

### Assessing the spatial extent of multiperil pest risk

Based on prior work by GEMS Informatics personnel and collaborators over the past decade, we assembled and spatially concorded global maps of the climate suitability of a given locale to sustain each of the following five fungal wheat diseases: stem, stripe and leaf rust, fusarium head blight (FHB), and septoria tritici blotch (STB). Specifically, we drew on previously reported CLIMEX models for four fungal diseases, including stem rust (Pardey et al., 2013), stripe rust (Beddow et al., 2015), leaf rust (Chai et al., 2020), and FHB (Turkington et al., 2016). Additionally, a CLIMEX model for STB was developed under the auspices of the HarvestChoice initiative (see, e.g., Koo and Pardey, 2020). All five CLMEX model outputs used for this study are openly accessible on the Data Repository for U of MN (DRUM) system (see Data Availability). CLIMEX models map the suitability of the climate at a given locale to support the survival (for at least one generation) of each wheat pest (see, e.g., Sutherst and Maywald, 1985; Kriticos et al., 2015). The models represent each climate-suitable locale at 10 arc minute spatial grids (or 18.5 x 18.5 km pixel along the equator), for which we have 141,681 pixels representing the spatialized extent of wheat production worldwide sourced from Yu et al. (2020). We then calculated the extent of the spatial concordance of wheat area that was pest-free—in the sense that these pixels had climates considered unsuitable to sustain any of our five fungal diseases—and those pixels that may sustain at least one, two, three, and so on up to five of our target diseases. We report these as climate co-suitability counts to provide a straightforward indication of the multi-peril pest risk faced by wheat farmers around the world.

### Assessing the probabilistic consequences of multiperil crop losses

In this study, estimating the *probabilistic* losses worldwide associated with each of the five fungal pathogens, as well as the likely combined losses from all five, is conceptually equivalent to comparing a no-disease yield distribution with yield distributions under different disease pressure and management practices. Specifically, as illustrated in **Figure 2**, differences between the red vs green distributions represent the high-loss (counterfactual) regime where crop protection is absent, while differences between the blue vs green distributions represent the low-loss (counterfactual) regime where certain crop protection measures were implemented, albeit not all entirely effective in eliminating all pest-induced losses. Practical implementation of these complex counterfactual regimes is impaired by limited and incomplete data, but we assembled or created the best available data sets and proceeded as follows.

To develop our statistical representation of the yield loss *distributions* for each of the diseases we sourced data from the USDA-Cereal Disease Laboratory (2020) for stem, stripe, and leaf rust; for FHB we used Crop Protection Network (2020), supplemented with Nganje et al. (2004); and for STB we also sourced data from Crop Protection Network (2020). Using these loss data, we applied a maximum goodness-of-fit estimation method implemented in the fitdist function in R (Delignette-Muller and Dutang, 2015) to estimate the probabilistic beta-distribution of losses associated with each of these diseases. The resulting disease-specific beta distributions are plotted in **Supplementary Figure S1**, where the vertical axis indicates the likelihood of a given yield loss as revealed along the horizontal axis. The general shape, but notably not the position, of each distribution is similar, indicating a high frequency of little to low losses in a given year, and low odds of especially high yield losses.

### High-loss and low-loss regimes

*Stem rust loss distribution:* Following Pardey et al. (2013), we estimated two loss regimes for stem rust (caused by *Puccinia graminis*) to capture the high-loss distribution in the U.S. before 1960, a period subject to frequent stem rust epidemics, and a low-loss distribution in the U.S. spanning the post-1960 period when the deployment of resistant wheat varieties effectively mitigated crop losses associated with this pathogen.

*Stripe rust loss distribution:* In the U.S., losses from stripe rust (caused by *Puccinia striiformis f.* sp. *tritici*) were not significant prior to 1960 (Line, 2002; Beddow et al., 2015). From the 1960s to the early 2000s, stripe rust losses occurred mainly in the Pacific Northwest region of the U.S. Beginning around 2000, there appeared to be an increase in stripe rust losses in the central U.S. states and a spread into the (south) eastern part of the country (Beddow et al., 2015). For stripe rust, we estimated two yield loss distributions, one based on yield loss data before 1960 (designated a low-loss regime), and one based on yield loss data after 1960 (a high-loss regime).

*Leaf rust loss distribution:* Following Chai et al. (2020), there are no obvious shifts in the frequency and magnitude of losses due to leaf rust (caused by *Puccinia triticina*) in the U.S. over the past century. Thus, to estimate a high-loss distribution for leaf rust we used the yield loss data reported by USDA-CDL for the entire 1919-2020 time period. To construct a low-loss distribution, we drew all the observations from the subset of 1919-2020 losses that were below 1%, with the assumption that under a low-loss regime leaf rust losses would not exceed 1%.

*Fusarium head blight (FHB) loss distribution:* In the U.S., we obtained two sets of state-level yield loss data for FHB (caused by *Fusarium graminearum*). For the period 1993-2001, Nganje et al. (2004) reported state-level wheat FHB losses for nine FHB affected states growing three wheat classes (hard red spring, durum, and soft red winter), where major yield losses began in 1993 and continued through 2001. Beginning in 2018, the Crop Protection Network (2020) report wheat disease losses throughout the U.S. and Canada, from which we sourced state-level FHB yield losses for the period 2018-2020. In the U.S., FHB losses in wheat during the 1990s were comparatively high as a result of substantial FHB outbreaks (Nganje et al., 2004), and were thus used to construct our high-loss regime, while losses since 2018 were relatively low according to the Fusarium Head Blight newsletters from the U.S. Wheat & Barley Scab Initiative (USWBSI, 2022), and thus used to construct our low-loss regime.

*Septoria tritici blotch loss distribution:* The distribution of losses from septoria tritici blotch (caused by *Zymoseptoria tritici*, also known as *Mycosphaerella graminicola*) in the U.S. was estimated using state-level data for the 2018-2020 period reported by the Crop Protection Network (2021). State-level loss data for the entire 2018-2020 period were used to represent the low-loss regime because the U.S. losses reported for septoria tritici blotch during this period were generally low. We then used the subset of losses above 1% to represent a hypothetical high-loss regime.

### Probabilistic monte carlo simulations

Following Saari and Prescott (1985), we segmented the world into 15 epidemiological zones, with largely independent climatological patterns such that an epidemic in a given zone is likely to occur independently of epidemics in other zones (Beddow et al., 2013). Within each epidemiological zone, we geospatially intersected the HarvestChoice Spatial Allocation Model (SPAM) results for wheat with CLIMEX models for our targeted wheat fungal diseases to calculate the share of each zone, *z*, deemed suitable for wheat fungal disease *d* as


$$\beta_{x,d}=\frac{\left[\alpha_{z}^{\prime }(GI_{z,d,i}{}^{\circ}I_{z})–\alpha_{z}^{\prime }(GI_{z,d,n}{}^{\circ}(I_{z}-1))\right]}{\alpha_{z}^{\prime }1}$$

where ***α_z_*** is a vector of the total wheat production area in zone *z*’s cells; ***GI_z,d,i_*** is a vector of binary seasonal suitability indicators in zone *z* for disease *d* under an irrigation scenario (1 if suitable; 0 otherwise); ***GI_z,d,n_*** is a vector of binary seasonal suitability indicators in zone *z* for disease *d* under the non-irrigated scenario (1 if suitable; 0 otherwise); ***I_z_*** is a vector of binary indicators set to one if any wheat area in the corresponding cell was irrigated and zero otherwise; **1** is a vector of ones of the appropriate length, and “ ° “ is the operator indicating an element-by-element vector product.

With the disease-suitable area share *β_z,d_* identified, we then apply the loss distribution for each disease estimated above. Specifically, the proportional yield loss attributable to disease *d* can be represented by a random draw from their corresponding beta-distribution *l_d_* ~*Beta*(*a_d_*,*b_d_*) and the overall yield loss from a set of multiple wheat fungal diseases can be calculated as:


$$l_{z}=1–\prod_{d}\left(1-\beta_{z,d}l_{d}\right)$$

If the observed yield for zone *z* during year *t* is ${\mathrm{Yz}}_{,}^{\mathrm{ta}}$ and the total proportional yield loss from all wheat fungal diseases is *l_z,t_*, then the potential yield ${\mathrm{Yz}}_{,}^{\mathrm{tp}}$ (i.e., the counterfactual yield absent all wheat fungal diseases) can be calculated as:


$$Y_{z,t}^{p}=\frac{Y_{z,t}^{a}}{1-l_{z,t}}$$

The production losses for zone *z* and year *t* can then be calculated as:


$$L_{z,t}=(Y_{z,t}^{p}-Y_{z,t}^{a})A_{z,t}$$

where *A_z,t_* is the wheat production area in zone *z* and year *t*.

Using projected zonal wheat production for the period 2010-2050 from the International Agricultural Prospects (*i*AP) model (Pardey et al., 2014), we applied two alternative loss regimes with different combinations of loss distributions. Using this procedure, we obtain probabilistic estimates of the global production losses attributable to the five wheat fungal diseases during the period 2020-2050 benchmarked on the two alternative loss regimes.

### Economically justified R&D investment

To estimate the economically justifiable investment in R&D focused on avoiding the long-run crop losses associated with these wheat fungal diseases, we drew on the modified internal rate of return (MIRR) concept presented by Hurley et al. (2014). Specifically, we estimated the annual investment in R&D for the period 1990-2050 that achieved at least a 10% MIRR per year with a probability of 95%. Mathematically, we find the value *V* that solves:


$$V(i^{r},i^{c},i^{m})=max\left\{v:Pr\left(\sqrt[{60}]{\frac{\sum_{t=2000}^{2050}p_{t}L_{t}^{R}{\left(1+i^{r}\right)}^{2050-t}}{\sum_{t=1990}^{2050}v{\left(1+i^{c}\right)}^{1990-t}}}-1\geq i^{m}\right)>0.95\right\}$$

where *p_t_* is the price of wheat in year *t*, *i^r^* is the reinvestment rate, *i^c^* is the cost of capital, and *i^m^* is the modified internal rate of return (MIRR). Here, we assume a 10-year research adoption lag to account for the latency in the adoption of new rust resistant wheat varieties. This equation calculates the annual investment amount for 1990 to 2050 that yields a MIRR of at least *i^m^* (targeting 10 percent per year) with a probability of 0.95. Based on the MIRR method in Pardey et al. (2013), the underlying reinvestment rate *i^r^* is taken to be 3 percent per year and the cost of capital rate *i^c^* is set at 10 percent per year.

## Data availability statement

Publicly available datasets were analyzed in this study. This data can be found here: The CLIMEX model outputs used for all five wheat fungal diseases are openly accessible through the Data Repository for U of MN (DRUM) system at https://doi.org/10.13020/0QSZ-W114; Yield losses from rust diseases in the U.S. were downloaded from https://www.ars.usda.gov/midwest-area/stpaul/cereal-disease-lab/docs/small-grain-losses-due-to-rust/small-grain-losses-due-to-rust/; Yield losses in the U.S. for other wheat diseases were sourced from https://loss.cropprotectionnetwork.org/crops/wheat-diseases; Other datasets generated during and/or analyzed as part of the current study are maintained on the GEMS Informatics Platform and can be requested from the corresponding author YC for use to replicate the findings of this study.

## Author contributions

YC and PP co-led and designed the study. SS and YC compiled and processed the CLIMEX models for wheat fungal diseases. YC performed the numerical analyses. YC, PP, and DH contributed to the writing. All authors contributed to the article and approved the submitted version.

## Funding

This work received funding support from 2Blades, the University of Minnesota’s GEMS Informatics Center, and CIMMYT, the International Maize and Wheat Improvement Center. Prior work on the development of CLIMEX models received support from the International Science and Technology Practice and Policy (InSTePP) Center at the University of Minnesota and the Bill and Melinda Gates Foundation Grant # OPPGD1450 by way of the HarvestChoice initiative.

## Acknowledgments

We thank Michael Kelleher from the 2Blades for his comments and edits. We thank Connie Chan-Kang of the GEMS Informatics Center for her research assistance.

## Conflict of interest

The authors declare that the research was conducted in the absence of any commercial or financial relationships that could be construed as a potential conflict of interest.

## Publisher’s note

All claims expressed in this article are solely those of the authors and do not necessarily represent those of their affiliated organizations, or those of the publisher, the editors and the reviewers. Any product that may be evaluated in this article, or claim that may be made by its manufacturer, is not guaranteed or endorsed by the publisher.
