# Supplementary material for: Multi-peril pathogen risks to global wheat production: A probabilistic loss and investment assessment
Source: Front Plant Sci. 2022 Oct 31;13:1034600. doi: 10.3389/fpls.2022.1034600 (PMC9659964; doi:10.3389/fpls.2022.1034600)
Supplement: Supplementary file 1 [file DataSheet_1.pdf]

Supplementary Material

Multi-Peril Pathogen Risks to Global Wheat Production: A Probabilistic Loss and Investment Assessment

Yuan Chai[1,2*], Senait Senay[2,3], Diana Horvath[4] and Philip Pardey[1,2]

[1]Department of Applied Economics, University of Minnesota, St. Paul, MN, United States

[2]GEMS Informatics Center, University of Minnesota, St. Paul, MN, United States

[3]Department of Plant Pathology, University of Minnesota, St. Paul, MN United States

[4]2Blades, Evanston, IL, United States

*** Correspondence:**
chaix026@umn.edu

Global Wheat-Related R&D Expenditure Estimates

Absent published data on global public wheat-related R&D expenditures, we used the following multi-step procedure to derive an estimate of public R&D spending on pest and disease related wheat R&D worldwide. Here pest and disease research includes all the crop breeding, plant pathology, entomology and related R&D within wheat research as encompassed in the USDA-CRIS (1993) Research Problem Areas (RPA) with designations "Control of Insects, Mites, Snails, and Slugs Affecting Field Crops and Range" and "Control of Diseases and Nematodes of Field Crops and Range" for the data covering the period 1970 to 1997, and USDA-CRIS (1998, 2005, 2013) Knowledge Areas with designations "Insects, Mites, and Other Arthropods Affecting Plants", "Pathogens and Nematodes Affecting Plants", "Diseases And Nematodes Affecting Plants", "Vertebrates, Mollusks, and Other Pests Affecting Plants", and "Biological Control of Pests Affecting Plants" for the data covering the period 1998 to 2018 .

Step 1

We first compiled a state-specific data series on U.S. public wheat R&D spending drawing on unpublished annual data files obtained from USDA-CRIS (various years) for the period 1980-2015. Wheat R&D spending concords with spending on research in the Research Problem Areas (RPA) and Knowledge Areas as described above. Together with the annual, state-level estimates of the wheat R&D expenditures incurred by the State Agricultural Experiment Stations (SAESs) maintained by the InSTePP Center at the University of Minnesota (see, e.g., Pardey and Smith 2017 and Pardey et al. 2016), we formed annual estimates of the intensity of wheat in total R&D (i.e., US public wheat R&D / US Public agR&D) for the period 1980-2015.

Using state-level estimates of the cash receipts by farmers for wheat sales taken from USDA-ERS (2020), and the total (farm level) value of cash receipts for all agricultural output taken from the same source, we formed annual, state-level estimates of the intensity of wheat in the total value of agricultural production, VOP (i.e., wheat VOP / ag VOP).

A linear regression was then run on the intensity of wheat R&D against the intensity of wheat value of production, forcing the regression through the origin such that

(*US Public Wheat R&D / US Public agR&D*) = 0.3923 (*US wheatVOP* / *US agVOP*),

with an $R^2 = 0.61$, and a standard error on the estimated coefficient equal to 0.0071. The state level intensities and fitted regression are plotted in Figure S3.

Step 2

Using a national level extraction of R&D data from (USDA-CRIS, various years) we estimated that the share of wheat pest and disease related R&D in total public wheat R&D averaged 25.5%, an average that was reasonably stable over the period 1970-2015.

Step 3

Assuming the estimated relationships in steps 1 and 2 are indicative of the structure of wheat R&D throughout the world, we formed estimates of the global wheat R&D spending and related pest and disease research as follows:

$$\text{Global wheat R\&D} \approx 0.3923 * \sum_i \text{Ag R\&D} * (\text{Wheat VOP} / \text{Ag VOP})$$

$$\text{Global wheat pest \& disease R\&D} \approx 0.255 * (\text{Global wheat R\&D})$$

where *i* indexes country. National wheat VOP and ag VOP values were obtained from FAO (2021).

The global wheat R&D and global wheat pest and disease R&D estimates were formed annually for the period 1980-2015 and expressed in 2018 PPP (purchasing power parity) dollars (World Bank 2020) that were obtained using country level implicit GDP deflator (UN, 2020). The $185 million and $540 million data reported in Figure 3 represent a simple average of the respective annual 1980-2015 series.

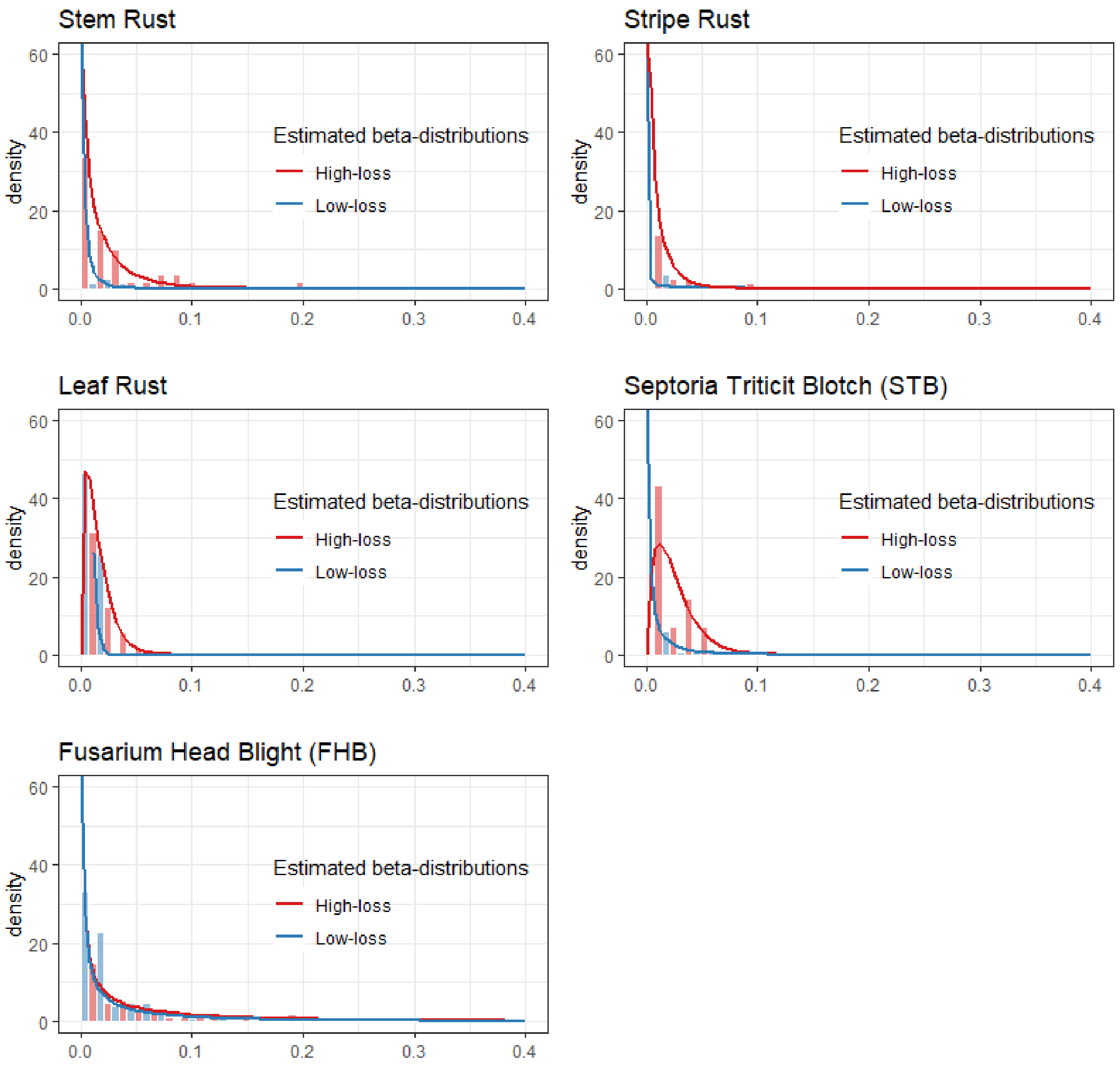

Supplementary Figure S1. The likelihood of wheat yield losses from fungal diseases

Notes: x-axis represents proportional yield loss reported for different wheat fungal diseases (e.g., 0.1 = 10% yield loss); y-axis represents the density (or likelihood) of different yield loss levels. The histograms represent observed yield loss data. The line plots represent the fitted beta-distributions.

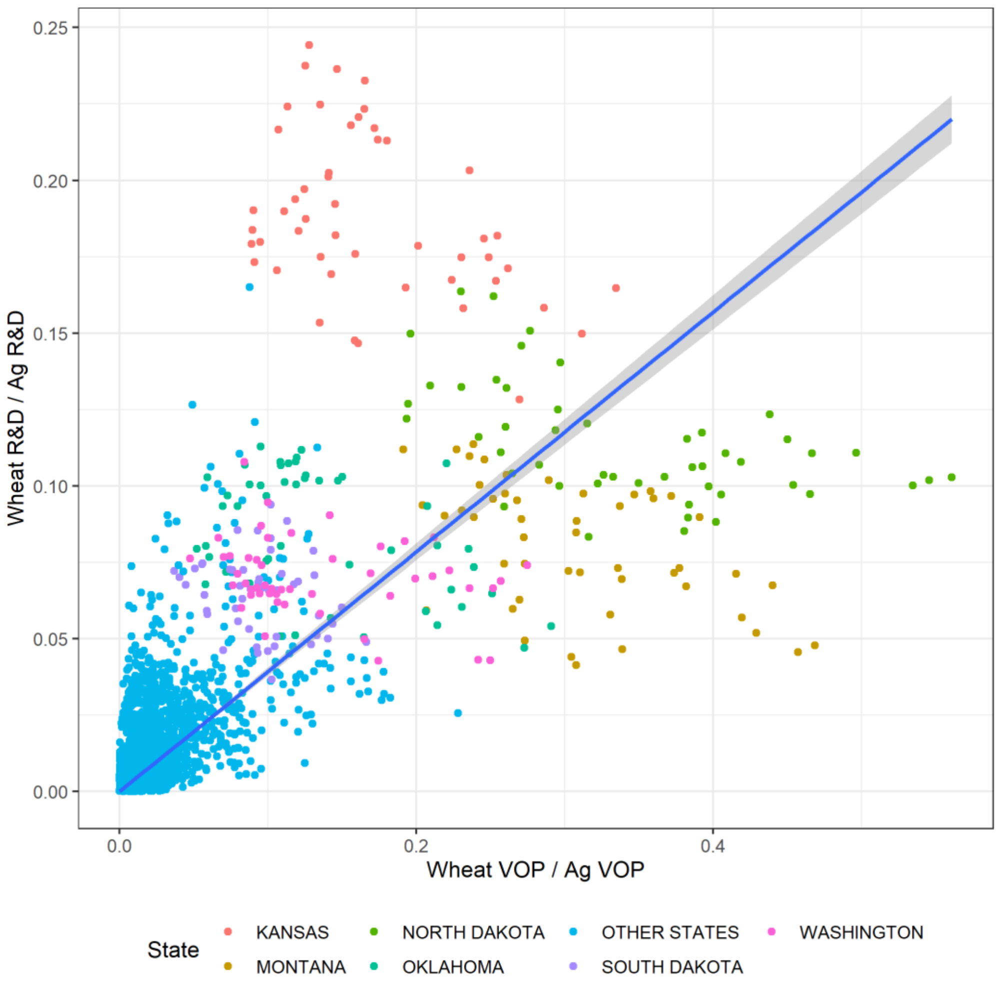

Supplementary Figure S2. The relationship between value of production and public R&D investments for US wheat

Supplementary Table S1. Regional multi-peril disease co-suitability

Region	Risk Free	Cumulative wheat area share co-suitable for multiple fungal diseases				
		**1**	**2**	**3**	**4**	**5**
				(percent)		
**Developed World**	**16.9**	**83.1**	**80.8**	**78.9**	**73.3**	**56.4**
Former Soviet Union	41.2	58.8	54.9	52.3	44.5	21.6
Western Europe	1.0	98.9	97.7	97.1	96.0	79.5
North America	4.5	95.5	95.2	92.0	86.7	82.2
Australasia	12.3	87.7	83.1	81.8	66.8	43.8
**Developing World**	**1.0**	**99.0**	**96.0**	**92.4**	**79.8**	**49.8**
Asia	0.9	99.1	95.9	92.0	77.9	45.1
Latin America	1.3	98.7	97.1	95.5	91.8	80.5
sub-Saharan Africa	2.9	97.1	94.9	93.1	90.4	74.5
**World**	**11.4**	**88.6**	**86.1**	**83.6**	**75.5**	**54.0**

Source: Developed by authors

[a] See Table 1 for details on regional groupings of countries.

References

FAO (Food and Agriculture Organization of the United Nations). 2021. FAOSTAT Statistical Database. Value of Agricultural Production. Rome, Italy: FAO. Downloaded from https://www.fao.org/faostat/en/#data/QV on December 21, 2021

Pardey, P.G., C. Chan-Kang, J.M. Beddow and S.M. Dehmer *Documentation: InSTePP International Innovation Accounts: Research and Development Spending, version 3.5 (Food and Agricultural R&D Series).* International Science and Technology Practice and Policy (InSTePP) Center, University of Minnesota, September 2, 2016. Available at https://www.instepp.umn.edu/products/documentation-instepp-international-innovation-accounts-research-and-development-spending.

Pardey, P.G. and V.H Smith. *Waste Not, Want Not—Transactional Politics, Research and Development and the US Farm Bill*. Policy Brief in the series "Agricultural Policy in Disarray, Reforming the Farm Bill." Washington D.C.: American Enterprise Institute, November 2017.

United Nations Statistics Division. UN National Accounts Main Aggregates Database. New York: United Nations, 2020. Downloaded February 4th 2020 from https://unstats.un.org/unsd/snaama/Downloads.

World Bank. "World Development Indicators Online" Washington, D.C.: World Bank, 2020b. Downloaded January 2020 from http://data.worldbank.org/data-catalog/world-development-indicators

USDA-CRIS (Current Research Information System). various years. Unpublished CRIS data files, authors extraction. Washington, DC: United States Department of Agriculture, Current Research Information System.

USDA-CRIS (Current Research Information System). 1993. *Manual of Classification of Agricultural and Forestry Research. Revision V. Classification used in the Current Research Classification System.* Washington, DC: United States Department of Agriculture, Current Research Information System.

USDA-CRIS (Current Research Information System). 1998. *Manual of Classification of Agricultural and Forestry Research. Revision VI. Classification used in the Current Research Classification System.* Washington, DC: United States Department of Agriculture, Current Research Information System.

USDA-CRIS (Current Research Information System). 2005. *Manual of Classification for Agricultural and Forestry Research, Education, and Extension. Revision VII. Classification used in the Current Research Classification System.* Washington, DC: United States Department of Agriculture, Information Systems and Technology Management.

USDA-CRIS (Current Research Information System). 2013. *Manual of Classification for Agricultural and Forestry Research, Education, and Extension. Revision VIII. Classifications used in the Research, Education, and Extension Project Online Reporting Tool (REEport).* Washington, DC: United States Department of Agriculture, National Institute of Food and Agriculture.

USDA-ERS (Economic Research Service). 2020. Agricultural Productivity in the United States. Washington, DC: United States Department of Agriculture. Farm Income and Wealth Statistics, Annual Cash Receipts by Commodity. Downloaded on December 23rd, 2020 from https://data.ers.usda.gov/reports.aspx?ID=17832.
